# The protective activity of natural flavonoids against osteoarthritis by targeting NF-κB signaling pathway

**DOI:** 10.3389/fendo.2023.1117489

**Published:** 2023-03-14

**Authors:** Yongjun Ye, Jianguo Zhou

**Affiliations:** ^1^ Department of Orthopedics, First Affiliated Hospital of Gannan Medical University, Ganzhou, China; ^2^ Department of Joint Surgery, Ganzhou People’s Hospital, Ganzhou, China

**Keywords:** flavonoids, osteoarthritis, NF-κB, inflammation, extracellular matrix (ECM)

## Abstract

Osteoarthritis (OA) is a typical joint disease associated with chronic inflammation. The nuclear factor-kappaB (NF-κB) pathway plays an important role in inflammatory activity and inhibiting NF-κB-mediated inflammation can be a potential strategy for treating OA. Flavonoids are a class of naturally occurring polyphenols with anti-inflammatory properties. Structurally, natural flavonoids can be divided into several sub-groups, including flavonols, flavones, flavanols/catechins, flavanones, anthocyanins, and isoflavones. Increasing evidence demonstrates that natural flavonoids exhibit protective activity against the pathological changes of OA by inhibiting the NF-κB signaling pathway. Potentially, natural flavonoids may suppress NF-κB signaling-mediated inflammatory responses, ECM degradation, and chondrocyte apoptosis. The different biological actions of natural flavonoids against the NF-κB signaling pathway in OA chondrocytes might be associated with the differentially substituted groups on the structures. In this review, the efficacy and action mechanism of natural flavonoids against the development of OA are discussed by targeting the NF-κB signaling pathway. Potentially, flavonoids could become useful inhibitors of the NF-κB signaling pathway for the therapeutic management of OA.

## 1 Introduction

Osteoarthritis (OA), a common joint disease characterized by low-grade chronic inflammation, often causes disability, decreases life quality, and increases social and economic burdens. OA greatly affects more than 250 million people around the world, and the prevalence is increasing, particularly among the elderly and obese (1). Pathologically, the alterations in joint tissues contribute to the development for OA, including inflammatory responses, marginal osteophyte formation, and subchondral osteosclerosis. Currently, most therapeutic pharmaceuticals for OA management are prepared for pain alleviation and symptom improvement rather than for OA prevention or cure. Surgery is typically considered the most effective management of knee OA (2). This might be attributed to the insufficient understanding of the pathological mechanisms of OA, which are orchestrated by imbalanced signaling networks. Several efforts have been made on many important signaling pathways, such as NF-κB (3), MAPK (4), Wnt/β-catenin (5), TGFβ/Smad (6), and BMP pathways (6). However, potential targets and pharmacologically effective drugs for OA management are still needed.

Chondrocytes, the unique cell type in joint cartilage, synthesize the extracellular matrix (ECM) and maintain the homeostasis of cartilage, which is an avascular tissue and has a limited repair capacity. However, chondrocytes are easily negatively affected by many detrimental stimuli, and dysregulated biological activities in chondrocytes may produce significant alterations in metabolism. Increased catabolism and decreased anabolism may lead to the degenerative development of OA. For example, increased catabolic activity in the ECM can be promoted by enhanced expression of matrix metalloproteinases (MMPs) and disintegrin and metalloproteinase with thrombospondin motifs (ADAMTSs), which mainly degrade the main components of type II collagen and aggrecan (7). Chronic inflammation has been implicated in the development of OA. Patients with OA are often observed with increased levels of pro-inflammatory cytokines, such as IL-1β and TNFα (8). Chondrocytes can be stimulated by the pro-inflammatory cytokines IL-1β, IL-6, and TNFα, promoting an imbalance in metabolism and leading to the pathological development of OA.

NF-κB signaling plays a crucial role in inflammatory responses, which contribute to chondrocyte cell death, ECM degradation, and cartilage destruction (9). Mechanically, NF-κB can act as a transcriptional factor to regulate the expression of pro-inflammatory cytokines. Thus, NF-κB signaling has become a potential target, and inhibition of NF-κB signaling can effectively ameliorate the pathological development of OA (10). Flavonoids, a class of natural polyphenolic compounds, are chemically marked by a 15-carbon (C_6_-C_3_-C_6_) skeleton with various substitutions. Although flavones and flavonols in the form of aglycone can be naturally obtained in a small amount, flavonoids in plants are generally maintained as glycosides by binding to sugars in the form of β-glycosides. The sugars attached to these flavonoids are generally D-glucose or L-rhamnose. Flavonoids in plants are associated with signaling pathways for defense (11). The glycoside forms of flavonoids have higher structural stability and water solubility. However, they exhibit relatively poor bioavailability. It has been demonstrated that glycated flavonoids can usually be hydrolyzed by gut microbiota or intestinal enzymes into aglycones, which are easier to absorb (12). After absorption, flavonoids may undergo conjugation. Hopefully, β-glucuronidase in the tissues may induce the release of active flavonoids (12).

**Figure 1:**
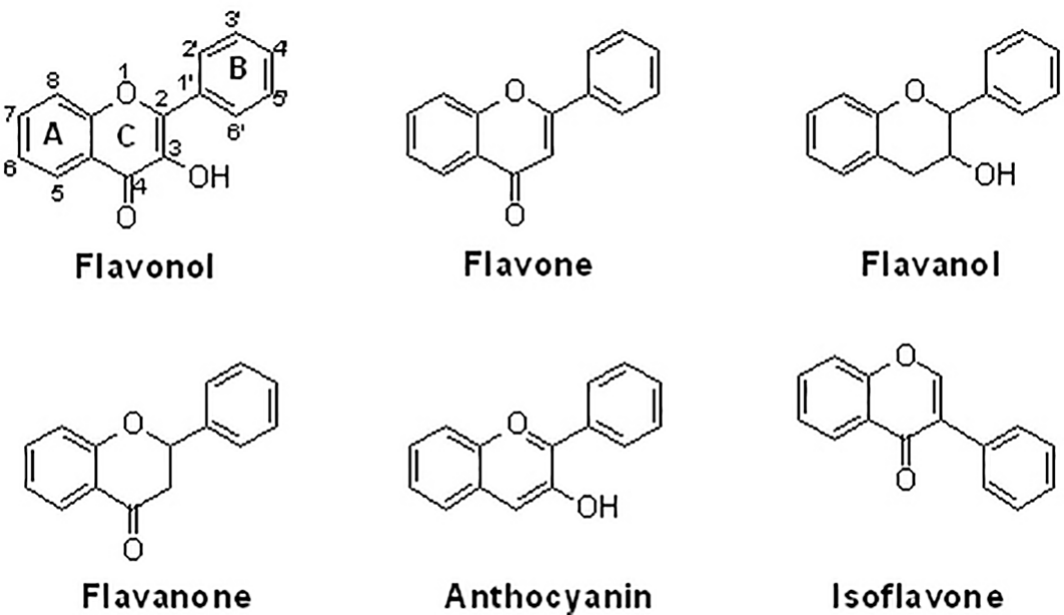
The chemical structures of six sub-groups of flavonoids. Natural flavonoids can be divided into flavonol, flavone, flavanol, flavanone, anthocyanin, and isoflavone.

Supplemental natural flavonoids as nutraceuticals are increasingly recognized for the management of many diseases, particularly those that are chronic. Consumption of natural flavonoids provides health-benefial effects on bone and cartilage diseases. Some flavonoids, particularly isoflavones, can function as phytoestrogens due to their structural similarity to estrogen and their ability to bind to estrogen receptors. The estrogen-like effects of flavonoids favor anabolism in the tissues of bone and cartilage, providing similar effects to hormone (13). Structurally, natural flavonoids can be divided into several sub-groups regarding the degree of oxidation in the central heterocycle, mainly including flavonols, flavones, flavanols/catechins, flavanones, anthocyanins, and isoflavones (14) (**Figure 1**). Natural flavonoids have been demonstrated to have various biological activities, including anti-inflammation, anti-oxidation, anti-cancer, and bone protection (15). For example, xanthohumol (40 mg/day by intragastric administration for 8 weeks in mice and 10, 25, and 50 μM for cell culture) may exhibit inhibitory activity against the production of inflammatory cytokines and the expression of ECM catabolic enzymes by upregulating NRF2 and downregulating NF-κB pathways *in vivo* and *in vitro* (16). The protective activity of flavonoids against OA development might be associated with the inhibition of NF-κB signaling (17). In this article, we will provide a comprehensive discussion in this field.

## 2 NF-κB signaling in the physiology and pathogenesis of chondrocytes

### 2.1 The biological functions and regulations of NF-κB signaling

**Figure 2:**
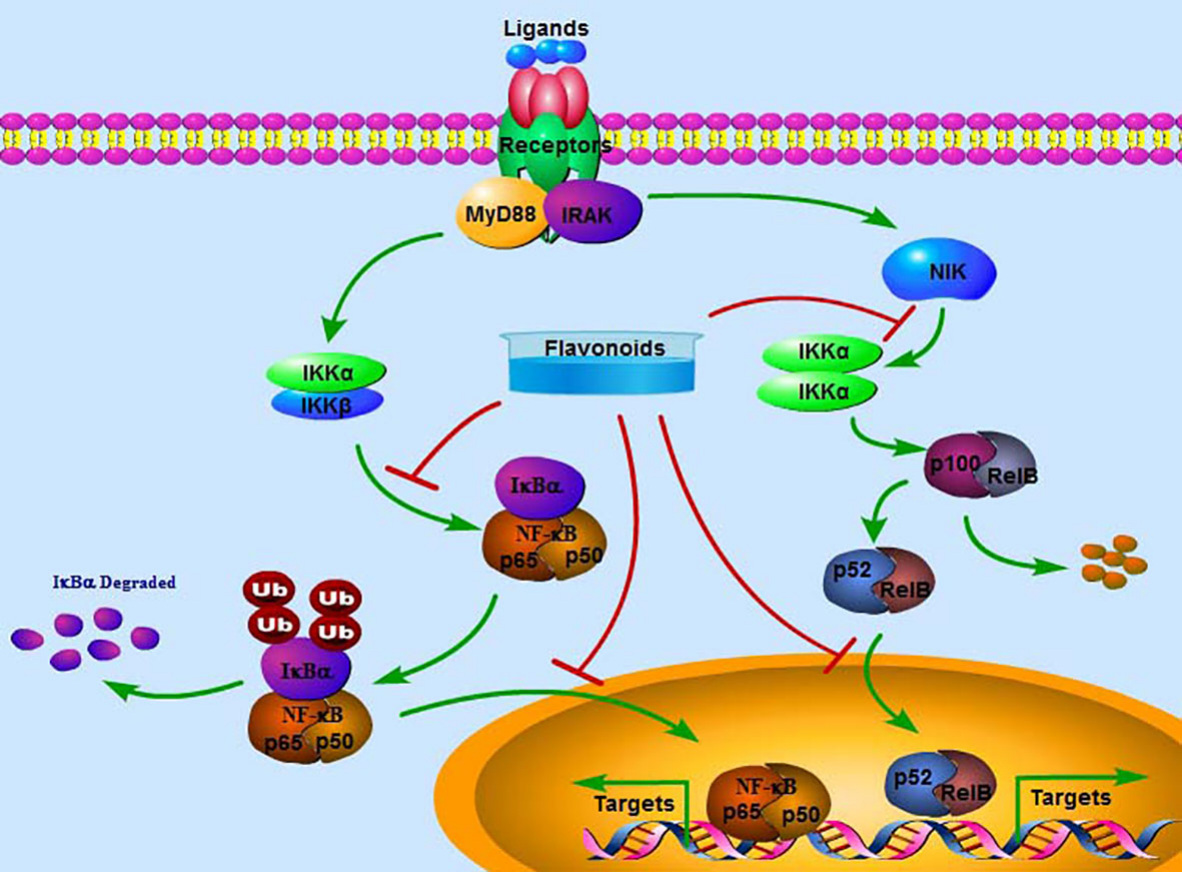
The classical signaling pathway of NF-κB. Extracellular ligands can activate the NF-κB pathway by interacting with the receptor, which activates and phosphorylates IKKα/β. Activated IKKα/β may phosphorylate and degrade IκBα, releasing p65 and p50. The complex p65/p50 enters the nucleus to transcriptionally regulate the expression of target genes. Alternatively, NIK-activated IKKα/α can further stimulate p100 to be processed into p52, which forms a complex with RelB and then enters the nucleus to transcriptionally regulate the expression of target genes. Flavonoids may inactivate the NF-κB pathway by inhibiting the phosphorylation of IKKα/β and IκBα, the nuclear translocation of p65/p50 and p52/RelB and suppressing the expression of target genes.

The NF-κB family of ubiquitously expressed transcriptional factors includes p65 (RelA), RelB, NF-κB1 (p105/p50), NF-κB2 (p100/p52), and c-Rel. All members have an evolutionarily conserved region: the N-terminal Ref-1-homology domain (RHD), which regulates dimerization, nuclear localization, DNA interaction, and association with related inhibitors. No transactivation domains in either NF-κB1 or NF-κB2 are observed. Therefore, homodimers or heterodimers between NF-κB1 and NF-κB2 cannot exhibit biological activity as transcriptional factors. Up to 15 different dimer combinations have been reported (18). Among them, the p65/p50 dimer is the most abundant and expressed in almost all cell lines (19). Under physiological conditions, the NF-κB dimers are inactivated by the inhibitory factor IκB and retained in the cytoplasm. Under stress conditions, active IκB kinase (IKKs) can phosphorylate IκB and induce its degradation *via* the ubiquitin-proteasome system, leading to the release of NF-κB dimers and translocation into the nucleus for transcriptional regulation of target gene expression (20) (**Figure 2**). Alternatively, NF-κB signaling can be activated by interacting with members of the TNF receptor superfamily, such as CD40, LTβR, and the receptor activator of NF-κB (RANK). P100 can be processed into p52 by NF-κB-inducing kinase (NIK) and IKKα, and p52 can form a heterodimer with RelB to be activated. Subsequently, the p52/RelB dimer translocates into the nucleus for transcriptional regulation. However, the specific binding sequences for p52/RelB have not been identified (21).

Activated NF-κB complexes in the nucleus may interact with the NF-κB response elements and then transactivate the expression of target genes, which include proinflammatory cytokines, chemokines, adhesion regulators, growth factors, and immunomodulatory proteins (20). NF-κB is also a critical transcriptional factor for sensing redox balance, and it can be stimulated or suppressed by reactive oxygen species (ROS) (22). In many cases, such as cytotoxicity and inflammation reactions, ROS can target and activate NF-κB. H_2_O_2_ is often used as a stimulator to produce ROS, and H_2_O_2_ may activate NF-κB. Interestingly, H_2_O_2_-activated NF-κB functions with different underlying mechanisms in specific cell types (23, 24). The biological activity of NF-κB can be mediated by posttranslational modifications, including methylation, acetylation, phosphorylation, and ubiquitination (25). For instance, histone deacetylase 5 (HDAC5) can induce deacetylation of p65 at lysine-310, leading to downregulation of the transcriptional activity of p65 (26). However, p65 phosphorylation at serine-276 may increase the acetylation of lysine-310 (27).

### 2.2 The pathogenesis of OA

It is well recognized that low-grade, chronic inflammation has been shown to play a central role in the development of OA, which is considered a complex, multifactorial joint pathology stimulated by inflammation and metabolic factors. The involvement of inflammation in the histopathological development of OA has been demonstrated since the early 1980s (28). Inflammatory cytokines have been shown to support inflammatory responses in both synovial cells and chondrocytes. The injured chondrocytes may produce damage-associated molecular patterns (DAMPs) in the cartilage tissues, leading to further enhancement of inflammatory processes in the synovial membrane, which secretes detrimental catabolic factors to increase chondrocyte damage with a feedback loop (29). Specifically, pro-inflammatory cytokines produced by chondrocytes have been demonstrated to attenuate anabolic activity, stimulate proteolytic enzymes, and promote ECM degradation and cartilage loss (30). An imbalance between anti-inflammatory and pro-inflammatory cytokines leads to catabolism. Several pro-inflammatory cytokines include IL-1β, IL-6, IL-18, TNFα, and leukemia inhibitory factors. IL-4, IL-10, TGFβ, and IFNγ are considered anti-inflammatory factors (31). Inflammasome activation is induced by secondary arthritis. Inflammasome-regulated self-activation of caspase-1 stimulates the proteolytic activation of the inflammatory factors IL-1β and IL-18, which are important members of the IL-1 family. Upon interacting with their specific receptors, these factors can transduce signals and activate NF-κB and p38 MAPK signaling pathways, which trigger the expression of IL-6, IL-8, and IFN-γ (32). Particularly, IL-1 and IL-18 may upregulate the expression of catabolic factors, such as MMPs (33). Many anti-inflammatory candidates, such as IL-1 inhibitor (canakinumab), TNFα inhibitor (adalimumab), and IL-6 inhibitor (tocilizumab), have been developed for the therapeutic management of OA and other inflammatory diseases (34).

### 23 The physiological roles of NF-κB signaling in chondrogenesis

The expression of NF-κB signaling has been shown in the four zones of growth plates, particularly in the resting and hypertrophic zones. This indicates that NF-κB signaling is implicated in cartilage development and endochondral ossification (35). Inhibition of NF-κB signaling by overexpressing the inhibitory factor IκBα can arrest limb outgrowth during the limb development of a chick (36). Growth hormone insulin-like growth factor-1 (IGF-1)-activated NF-κB signaling has been reported to facilitate chondrogenesis, promote chondrocyte proliferation and differentiation, and inhibit chondrocyte apoptosis. However, p65 siRNA transfection-induced inactivation of NF-κB signaling may reverse the effects of IGF-1 by downregulating the expression of the BMP2 pathway (37). Interestingly, there are binding sites for NF-κB in the BMP2 gene promoter, and NF-κB might induce chondrocyte proliferation and cartilage formation *via* upregulating the expression of BMP2. In cultured ATDC5 cells, knockdown of p65 can cause inhibition of Sox9 expression by downregulating BMP2 expression (38).

### 2.4 The roles of the classical NF-κB signaling in OA

In primary human OA chondrocytes, the expression of NF-κB signaling is activated (39). Knockdown of IKKβ has been reported to increase the deposition of collagen II in a SOX9-independent manner and decrease the expression of runt-related transcription factor 2 (RUNX2). A deficiency of IKKα enhances the production of glycosaminoglycan in a SOX9-dependent manner. Particularly, IKKβ knockdown can suppress the synthesis of IL-1β-induced MMP-13, which is a transcriptional target of NF-κB (39). Consistently, HIF-2α is also a transcriptional target of NF-κB, and increased expression of HIF-2α is positively correlated with OA development. The promoter activity of both MMP-13 and ColX has been reported to be increased by HIF-2α. NF-κB may promote the remodeling of cartilage tissues by mediating the expression of HIF-2α (3, 40). In addition, HIF-2α-regulated CCAAT/enhancer-binding protein β (C/EBPβ) also upregulates the expression of MMP-13 by enhancing the promoter activity of C/EBPβ (41). Many gene expressions of catabolic factors, such as MMP-1, MMP-9, ADAMTS-4, and ADAMTS-5 and pro-inflammatory mediators, such as COX-2, PGE2, and iNOS, are directly regulated by NF-κB (9, 42, 43). Collectively, NF-κB functions as a transcriptional factor to orchestrate the expression profiles of target genes, which are involved in ECM degradation and inflammation in the pathogenesis and progression of OA.

### 2.5 The roles of the alternative NF-κB signaling in OA

The alternative NF-κB pathway is also found to contribute to the development of OA (3, 44). The critical role of the alternative NF-κB pathway in maintaining bone homeostasis has been reported. In *p100* knockout mice, the number of osteoclasts is increased, and the number of osteoblasts is decreased (45). Particularly, the ankyrin repeats at the C-terminus of NF-κB2 are homozygously deleted in *p100*-knockout mice. The activity of Rel/NF-κB cannot be suppressed by p100, and the p52/RelB complex facilitates interaction with DNA (46). In addition, phosphorylated NF-κB2 and RelB are found to be active in chondrocytes in the periarticular zone of the growth plate but rarely in the hypertrophic zone. In *p100-*knockout chondrocytes, the alternative NF-κB pathway is constitutively activated. This may lead to the development of dwarfism and shortened long bones due to abnormal growth plates and decreased proliferative activity of chondrocytes. However, the *p100-*knockout-induced defect in the growth plate can be partially rescued by a *p100*/*RelB* double knockout (44). Consistently, the hypertrophic zone has been found to have increased thickness by two to three folds and increased expression of type X collagen in *p50/p52* double knockout mice (47).

### 2.6 The epigenetic regulation associated with NF-κB in OA

Epigenetic regulation of histones at the protein level is found in OA. The activity of NF-κB in OA chondrocytes can be regulated by HDACs, which exhibit deacetylation activity. It has been reported that acetylation of p65 facilitates its nuclear translocation (48). Recently, it has been reported that HDAC10 is highly related to the expression of IL-1β in synovium-derived mesenchymal stem cells (SMSCs) *in vivo* and *in vitro*. Overexpression of HDAC10 increases IL-1β-induced p-p65 and p65. Knockdown of HDAC10 may induce the retention of p-p65 in the cytoplasm and reduction in the nucleus (49). In OA fibroblast-like synoviocytes, HDAC inhibitors, such as SAHA (vorinostat) and LBH589 (panobinostat), can increase the binding activity of NF-κB to the promoter of miR-146a and negatively mediate IL-1β-induced pathways and cytokine secretion, displaying the potential rationale for anti-inflammatory activity (50). Consistently, SAHA has been shown to inhibit MMP-1, MMP-13, and iNOS expression by suppressing NF-κB nuclear translocation (51). In HDAC3-knockout chondrocytes, the acetylation of NF-κB is increased, and the expression of downstream factors, such as MMP-13, is also upregulated (52). However, one study reported that HDAC inhibitors do not affect the DNA-binding activity of NF-κB in human OA chondrocytes (53).

SIRT1, a nicotinamide adenine dinucleotide (NAD)-dependent nuclear histone deacetylase, has been reported to downregulate the activity of NF-κB in rat chondrocytes (54). SIRT1 promotes the deacetylation of p65 and suppresses the nuclear translocation of NF-κB, protecting against the inflammatory responses in articular chondrocytes and the development of OA (55). Overexpression of SIRT1 is associated with beneficial roles in OA, due to decreased acetylation of NF-κB, MMP-13, and ADAMTS-5 (56). SIRT1 expression exhibits protective activity against IL-1β-induced expression of cartilage-degrading enzymes, partially by inducing deacetylation of NF-κB, and it has become a potential therapeutic target for OA management (57). microRNAs (miRs) have been involved in the pathological development of OA. The association of miRs with the NF-κB pathway in OA chondrocytes has been discussed (58). miR-9 has been reported to reduce the production of pro-inflammatory cytokines, MMPs, and pro-apoptotic factors by targeting NF-κB in human articular chondrocytes (59). Overexpression of miR-326 can inhibit the expression of HDAC3, leading to increased acetylation of p65 and enhanced activity of STAT1 in chondrocytes (60). In addition, miR-30b-5p has been demonstrated to bind to the 3’-UTR of SIRT1, accompanied by enhancement of NF-κB activity and aggravation of articular cartilage loss and joint pain (61). Another study reported that transfection with miR-34a inhibitors may lead to a decreased level of p50 expression and nuclear translocation in human OA chondrocytes (62).

## 3 Natural flavonoids exhibit protective activity against OA development by inhibiting NF-κB signaling

Natural flavonoids have been implicated in protection against bone diseases due to their anti-inflammatory, anti-oxidative, and anti-apoptotic activities. Dietary interventions with polyphenols against OA, from preclinical to randomized clinical studies, have been discussed (63). Generally, natural flavonoids can attenuate the synthesis of important inflammatory cytokines, such as IL-6, TNFα, and PGE2, which contribute to the pathological development of OA. NF-κB signaling pathway has been demonstrated to orchestrate inflammatory responses and promote the expression of catabolic factors, such as MMPs and ADAMTSs (64). Flavonoids are reasonably effective for the therapeutic management of OA.

### 3.1 The different types of flavonoids

#### 3.1.1 Flavonols

**Figure 3:**
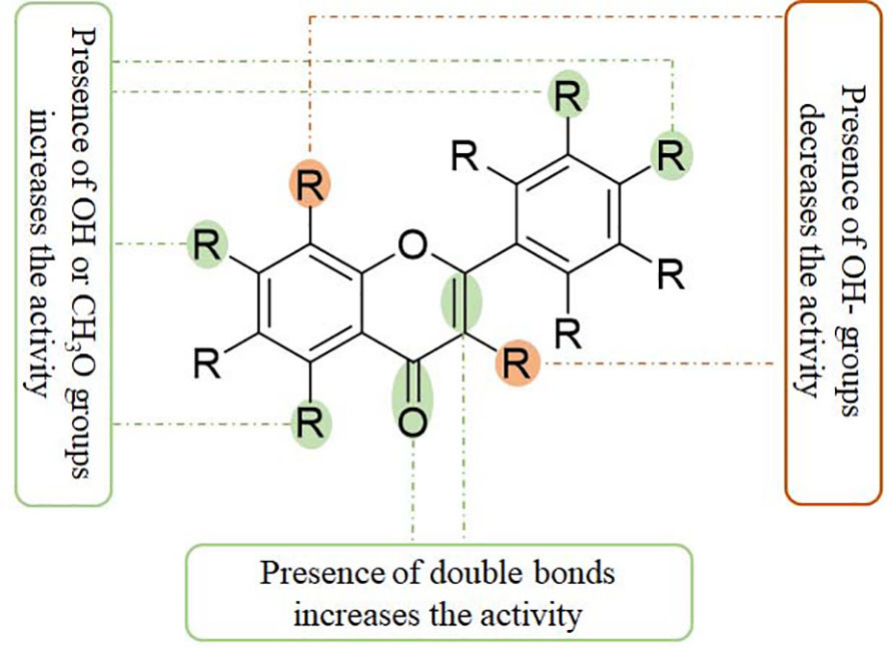
The structural relationship between flavonoids and anti-inflammatory activity. Presence of C2=C3 and C4=O double bonds may increase the anti-inflammatory activity. The presence of OH and CH3O groups at C-5, C-7, C-3’, and C-4’ positions also increase the anti-inflammatory activity. However, the presence of OH groups at C-3 and C-8 positions decreases the anti-inflammatory activity.

Myricetin and its glucoside form, myricitrin (also named myricetin-3-O-rhamnoside), often found in vegetables, tea, and berries, have been reported to decrease the production of inflammatory cytokines. In primary human chondrocytes, myricetin can lower the levels of TNFα, IL-6, NO, and PGE_2_, decrease the expression of COX-2, iNOS, MMP-13, and ADAMTS-5, and suppress the activity of NF-κB signaling, protecting against cartilage degradation. In addition, myricetin stimulates the expression of NRF2/HO-1 and PI3K/AKT signaling pathways (65, 66) (**Figure 3**). Similarly, kaempferol and its glucoside forms, such as astragalin (kaempferol 3-O-glucoside) and juglanin (kaempferol 3-O-arabinoside) can decrease the production of NO/iNOS and PGE_2_/COX-2 and inhibit the phosphorylation of IκBα and p65 in rat chondrocytes, ameliorating inflammation and protecting against OA development (67–69) (**Table 1**).

**Table 1:** Various flavonoids show protective activity against OA.

Structures	Models	Concentrations and routes	Biological activities	Ref.
Flavonols				
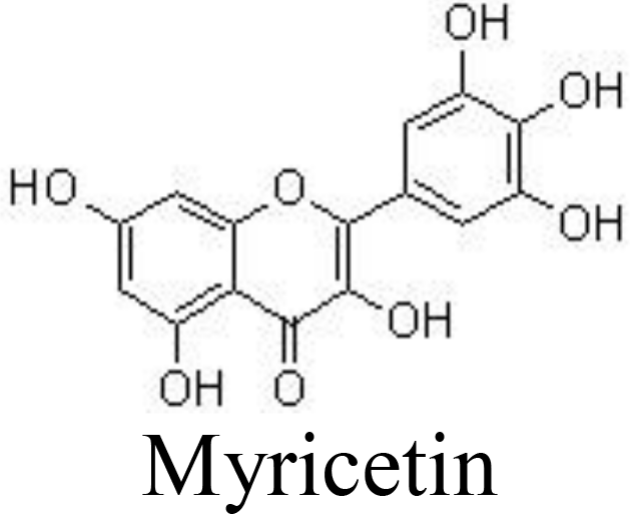 Myricetin	Primary human chondrocytes	5, 10, 15 μM	Decreases COX-2, iNOS, MMP-13, and ADAMTS-5; inhibits NF-κB signaling; stimulates NRF2/HO-1 and PI3K/AKT pathways.	Pan et al. (65)
	DMM-induced mouse OA	20 mg/kg every 2 days for 8 weeks; intragastric		
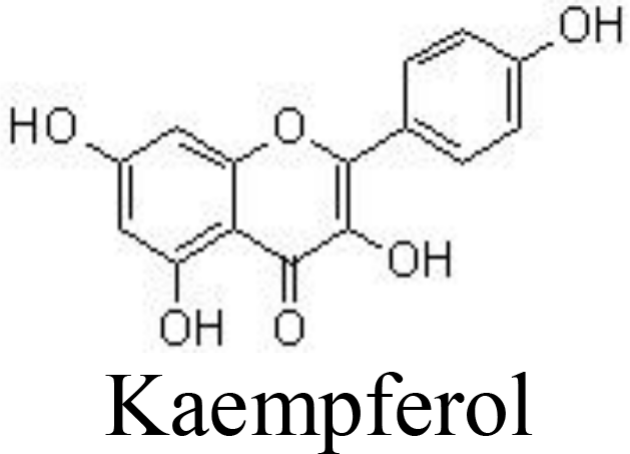 Kaempferol	Rat chondrocytes	25, 50, 100 μM	Decreases NO/iNOS, PGE_2_/COX-2. Inhibits IκBα and p65 phosphorylation. Activates PPARγ	Zhuang et al. (68)
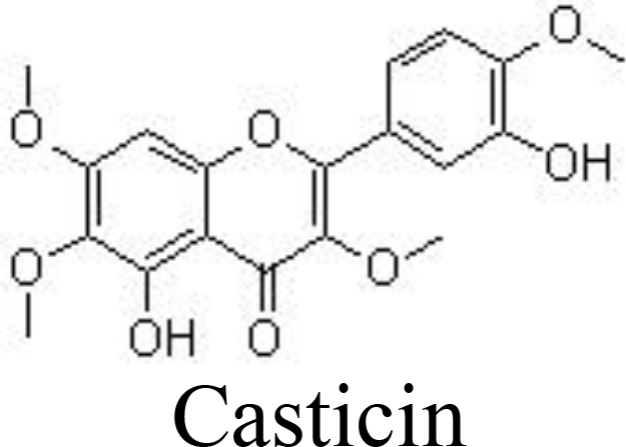 Casticin	ADTC5 cells	10, 20, 30 μM	Decreases PGE_2_, IL-6, and TNFα; Decreases ROS and MDA, increases SOD and GSH/GSSH; Inhibits MMP-3/-13, ADAMTS-4/-5. Inhibits IκBα and p65 phosphorylation	Mu et al. (70); Chu et al. (71)
	Human chondrocytes	6.25, 12.5, 25 μM		
	DMM-induced mouse OA	10 mg/kg every 2 days for 8 weeks; intraperitoneal injection		
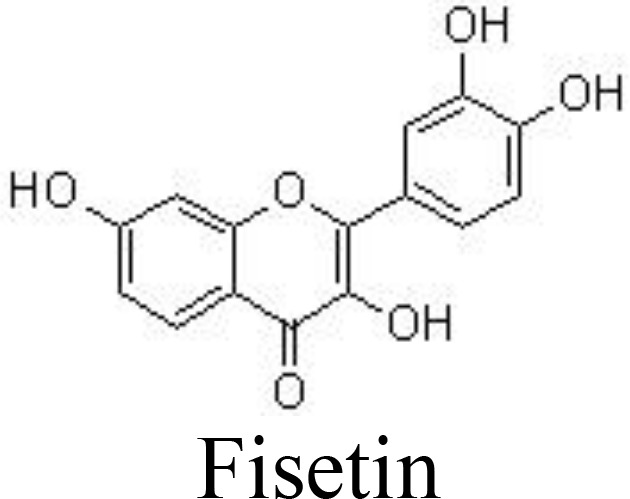 Fisetin	Primary human chondrocytes	1, 5, 10 μM	Decreases NO/iNOS, PGE_2_/COX-2, IL-6 Inhibits MMP-3, MMP-13, and ADAMTS-5 Inhibit collagen II and aggrecan degradation	Zheng et al. (72)
	DMM-induced mouse OA	20 mg/kg/day for 8 weeks; gavage		
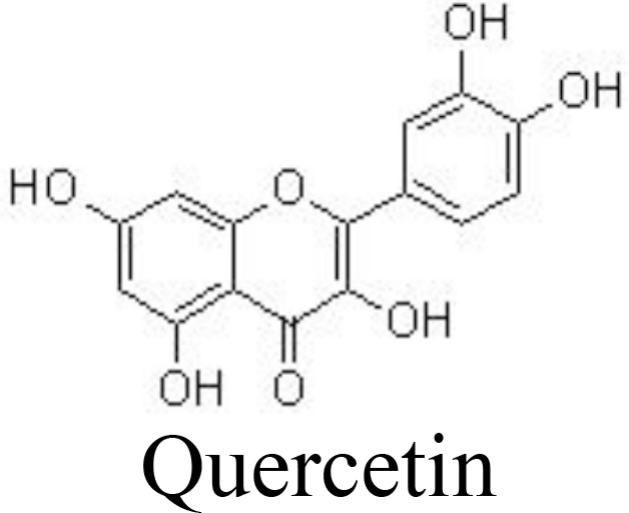 Quercetin	Papain-induced rat OA	1, 5, 10 mg/kg/day for 14 days; gavage	Ameliorates histopathological changes Decreases serum IL-1β and TNFα Inhibits TLR4 and NF-κB activity	Zhang et al. (73)
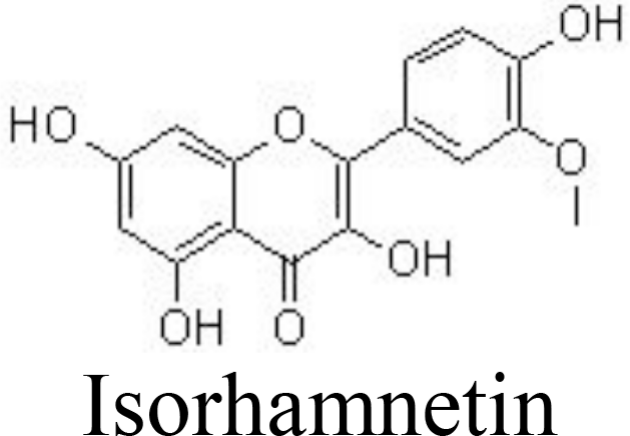 Isorhamnetin	Primary human OA chondrocytes	10, 50, 100 μg/mL	Decreases NO/iNOS and PGE2/COX-2 Inhibits stromelysin-1 and collagenase 3 Reduces ROS production Inhibits NF-κB, MAPK, and AKT pathways	Ji et al. (74); Zhou et al. (75)
	Mice chondrocytes	6.25, 12.5, 25 μM		
	ACLT-induced mouse OA	10, 20, 40 mg/kg every 2 days for 4 weeks; intraperitoneal injection		
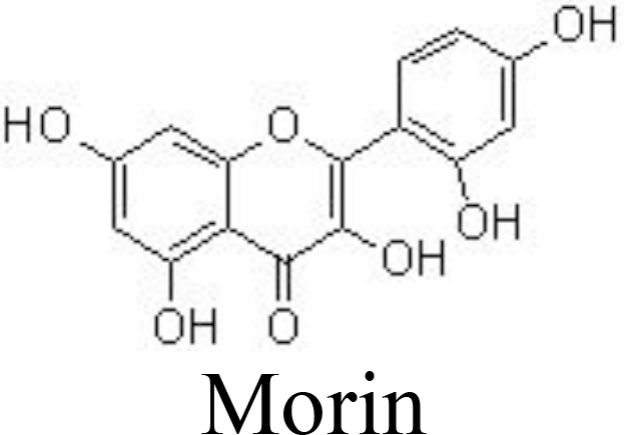 Morin	Primary human OA chondrocytes	1, 10, 50 μM	Decreases NO/iNOS and PGE2/COX-2 Inhibits NF-κB pathway	Chen et al. (76)
	IL-1β-induced rat OA	50 μM; joint cavity injection		
Flavones				
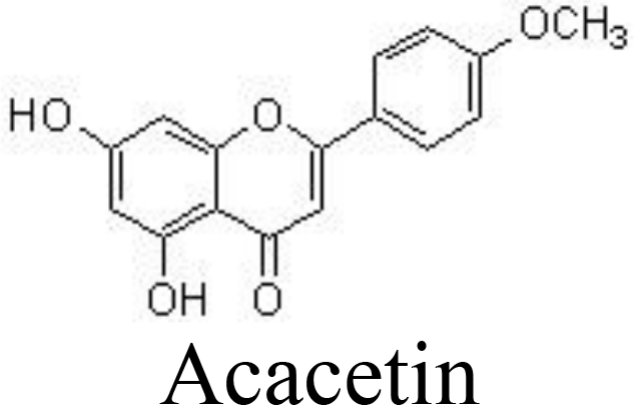 Acacetin	Primary mouse chondrocytes	3.125, 6.25 μM	Inhibits MMP-1, MMP-13, and MMP-13 expression *in vivo* and *in vitro*. Inhibits the degradation of IκBα. Lower OARSI scores.	Chen et al. (77)
	ACLT-induced mouse OA	3.125, 6.25 μM; joint cavity injection		
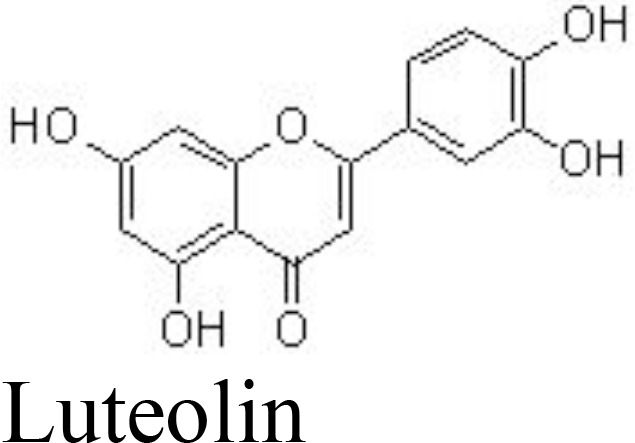 Luteolin	Rat chondrocytes	25, 50, 100 μM	Decreases NO/iNOS, PGE_2_/COX-2, TNFα, MMP-1/-2/-3/-8/-9/-13; Increases collagen II production; Inhibits p65 phosphorylation	Fei et al. (78)
	MIA-induced rat OA	10 mg/kg/day for 45 days; gavage		
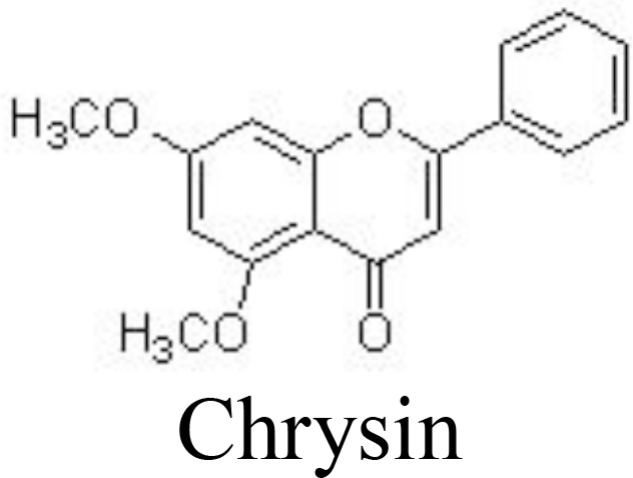 Chrysin	Human OA chondrocytes	1, 5, 10 μM	Reduces NO/iNOS, PGE2/COX-2, MMPs, and ADAMTSs; Increases aggrecan and collagen II; Inhibits IκBα phosphorylation	Zheng et al. (79)
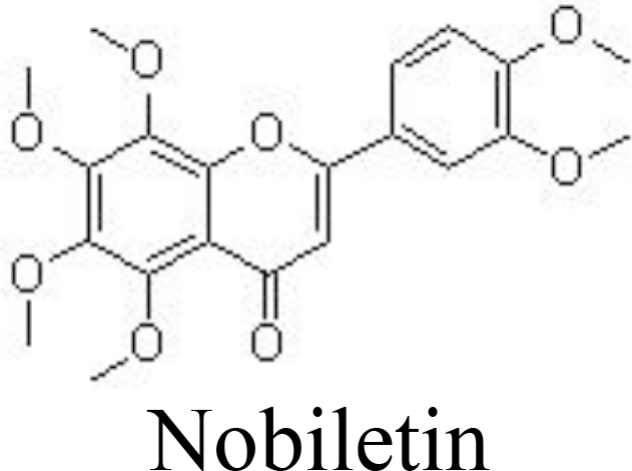 Nobiletin	Human OA chondrocytes	20, 40, 80 μM	Reduces NO/iNOS, PGE2/COX-2, MMP-13, and ADAMTS-5; Increases aggrecan and collagen II; Inhibits PI3K/AKT and NF-κB pathways	Xie et al. (80)
	DMM-induced mouse OA	20 mg/kg/day for 8 weeks; gavage		
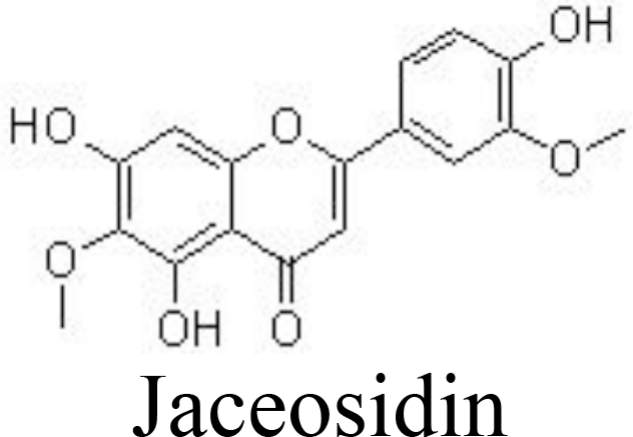 Jaceosidin	Primary mouse chondrocytes	20, 40, 80 μM	Decreases MMP-3/-13 and ADAMTS-4/-5 Inhibits NF-κB and MAPK pathways	Lee et al. (81)
	DMM-induced mouse OA	10, 25, 50 mg/kg every 2 days for 10 weeks; gavage		
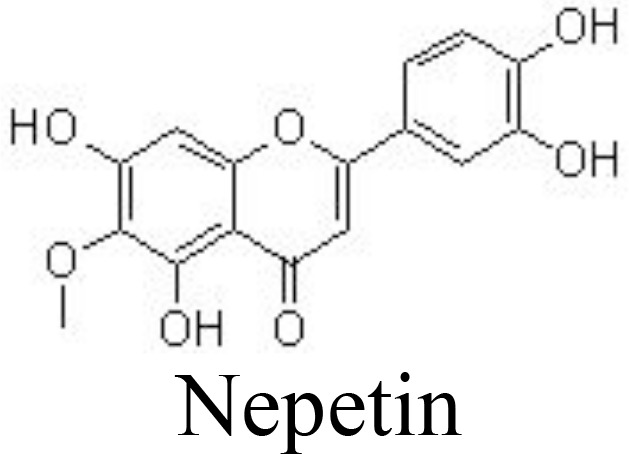 Nepetin	Primary mouse chondrocytes	2.5, 5, 10 μM	Decreases NO/iNOS, PGE_2_/COX-2, TNFα Inhibits MMP-1/-3/-13 and ADAMTS-4/-5 Inhibits NF-κB pathway by binding to p65	Xu et al. (82)
	DMM-induced mouse OA	20 mg/kg/day for 14 days; gavage		
Flavanols				
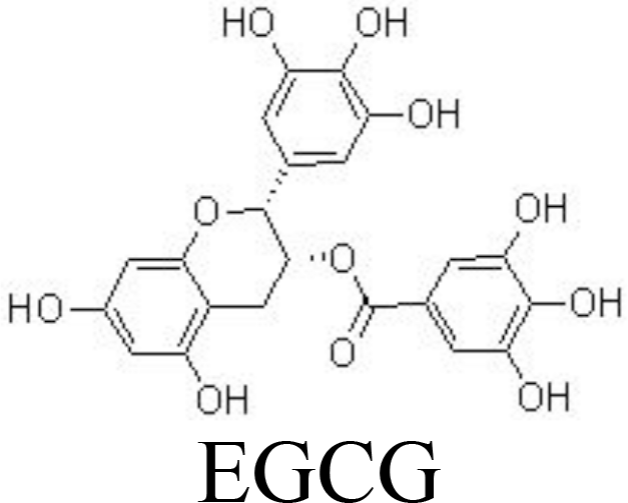 EGCG	Primary human OA chondrocytes	100 μM	Inhibits ENA-78, GM-CSF, GRO, GROα, IL-6/-8, MCP-1/-3, MIP-3α, MIP-1β, GCP2, IP-10, NAP-2, and LIF; Inhibits NF-κB and JNK/MAPK pathways	Akhtar and Haqqi (83)
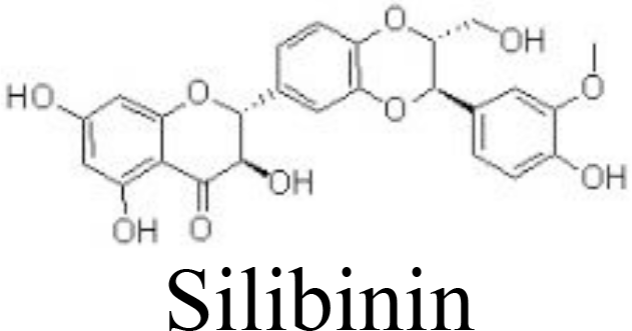 Silibinin	Primary human OA chondrocytes	1, 5, 10 μM	Decreases NO/iNOS, PGE_2_/COX-2, TNFα Inhibits MMP-1/-3/-13 and ADAMTS-4/-5 Inhibits PI3K/AKT and NF-κB pathways	Zheng et al. (84)
	DMM-induced mouse OA	200 mg/kg/day for 8 weeks; gavage		
Flavanones				
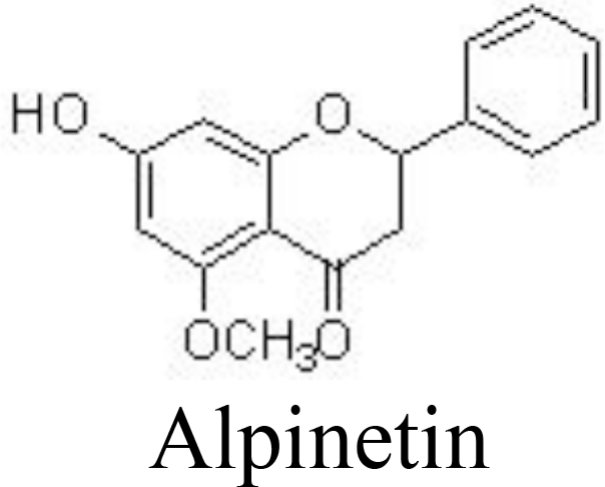 Alpinetin	Primary rat chondrocytes	10, 20 μM	Decreases MMP-13 and ADAMTS-5; Increases Col2a1, Bcl-2, and CKD1; Inhibits IκBα phosphorylation and p65 nuclear translocation; Stimulates ERK1/2 phosphorylation	Gao et al. (85)
	DMM rats OA	1 mM daily for 4 days, then every 2 days for another 10 days; joint cavity injection		
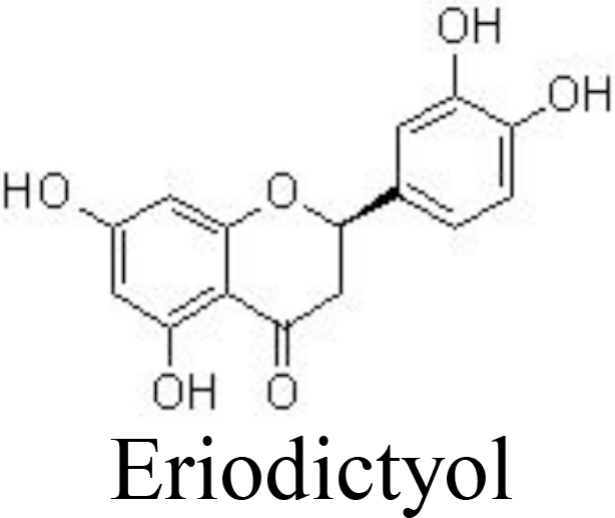 Eriodictyol	Primary human OA chondrocytes	6.25, 12.5, 25 μM	Decreases NO/iNOS, PGE_2_/COX-2, MMPs; Inactivates NF-κB pathway Activates NRF2/HO-1 pathway	Wang et al. (86)
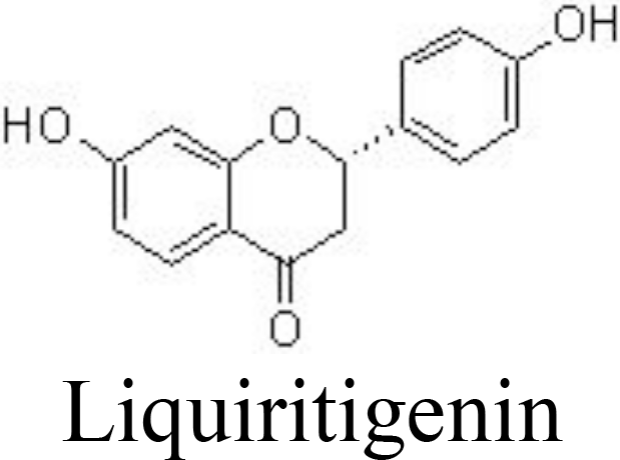 Liquiritigenin	Rat chondrocytes	20, 40 μM	Decreases NO/iNOS, PGE_2_/COX-2, MMPs; Inactivates NF-κB pathway	Tu et al. (87)
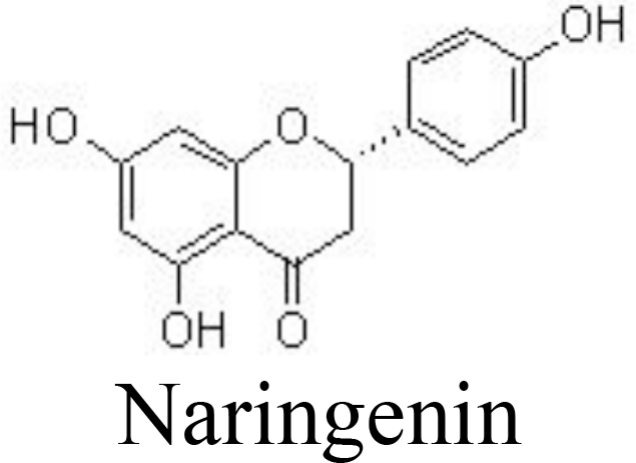 Naringenin	Primary rat chondrocytes	20, 40 μM	Reduces joint pain Suppresses MMP-1/-3/-13 and ADAMTS-4/-5; Inhibits NF-κB activation	Wang et al. (88)
	MIA-induced rat OA	20, 40 mg/kg/day for 2 weeks; gavage		
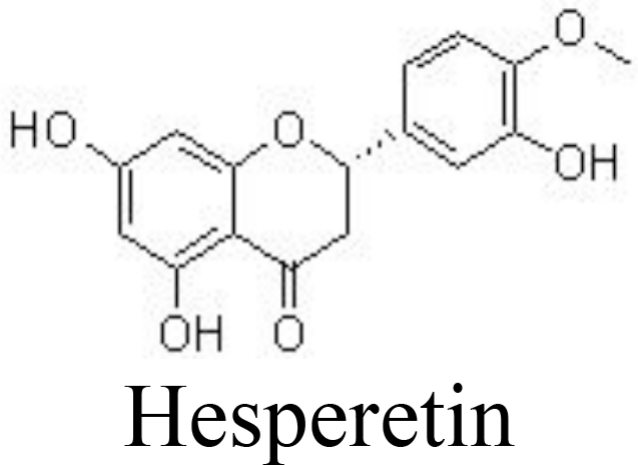 Hesperetin	Primary human chondrocytes	10, 20, 40 μM	Inhibits NO/iNOS, PGE_2_/COX-2, IL-6, TNFα, MMP-13, and ADAMTS-5; Suppresses NF-κB pathway Stimulates NRF2 pathway	Lin et al. (89)
	DMM-induced mouse OA	10 mg/kg/day for 8 weeks; intraperitoneal injection		
Anthocyanins				
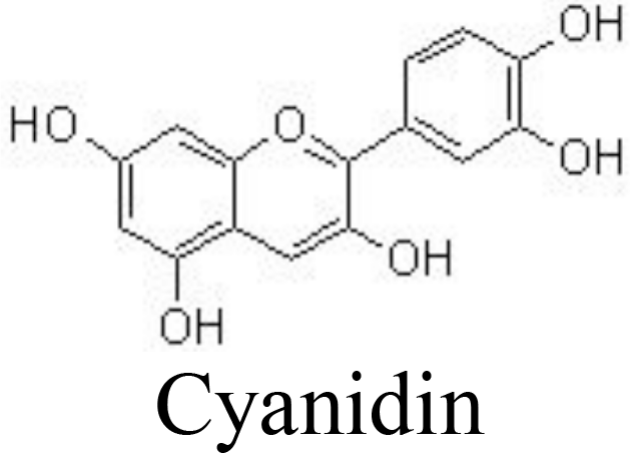 Cyanidin	Human OA chondrocytes	12.5, 25, 50 μM	Suppresses NO/iNOS, PGE_2_/COX-2, IL-6, TNFα, MMP-13, and ADAMTS-5; Enhances aggrecan and collagen II; Increases Sirt6 and inhibits NF-κB	Jiang et al. (90)
	DMM-induced mouse OA	50 mg/kg/day for 8 weeks; intragastric		
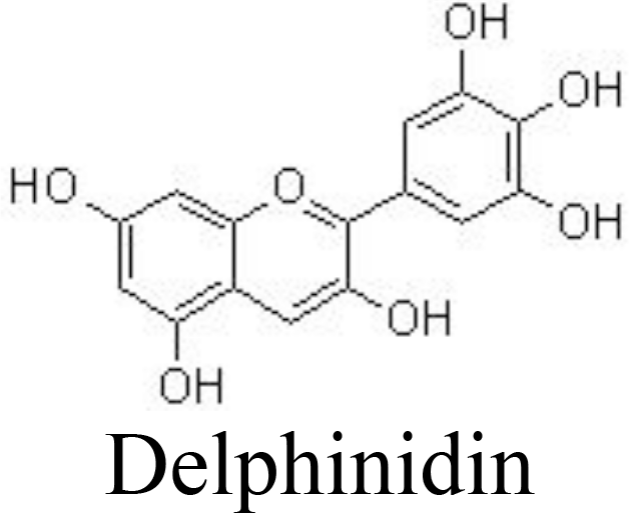 Delphinidin	Human OA chondrocytes	10, 50, 100 μg/mL	Decreases COX-2/PGE_2_ productions; Inhibits IRAK1^Ser376^ phosphorylation and NF-κB activation	Haseeb et al. (91)
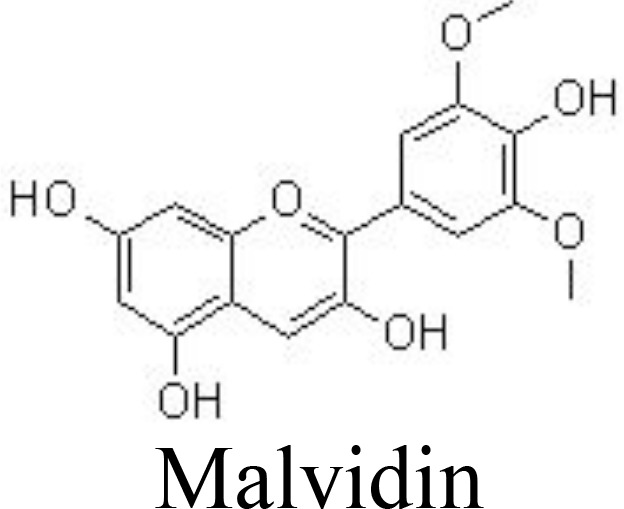 Malvidin	Primary rat chondrocytes	10, 20 μM	Relieves joint pain; Inhibits β-galactosidase expression Decreases IL-1β, IL-6, TNFα, and MMPs Inactivates NF-κB pathway	Dai et al. (92)
	MIA-induced rats OA	10, 20 mg/kg/day for 2 weeks; gavage		
Isoflavones				
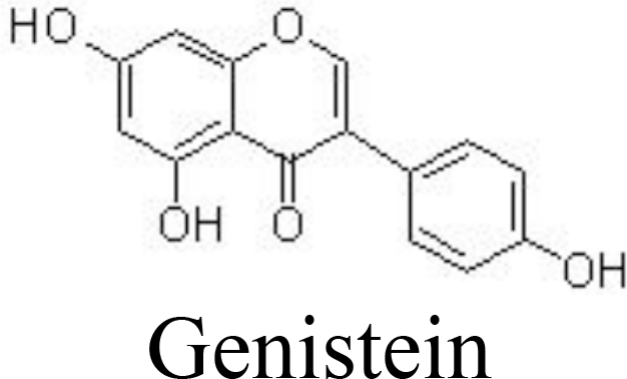 Genistein	Collagenase-induced TMJOA	30, 180 mg/kg/day for 4 weeks; intragastric	Improves the histopathological changes Reduces the levels of IL-1β and TNFα Inhibits the expression of p65	Yuan et al. (93)
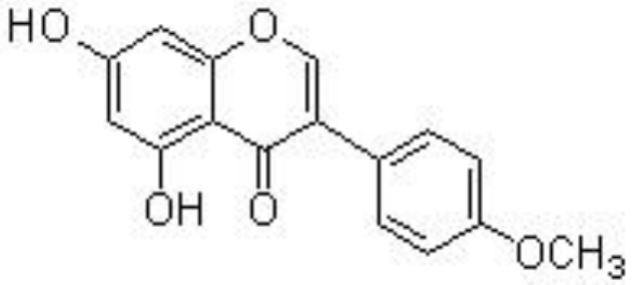 Biochanin A	Rat chondrocytes	7.5, 15 μM	Suppresses NOS-2 and COX-2/PGE2 Inhibits MMP-1/-3/-13 and ADAMTS-5 Inhibits NF-κB signaling	Oh et al. (94)
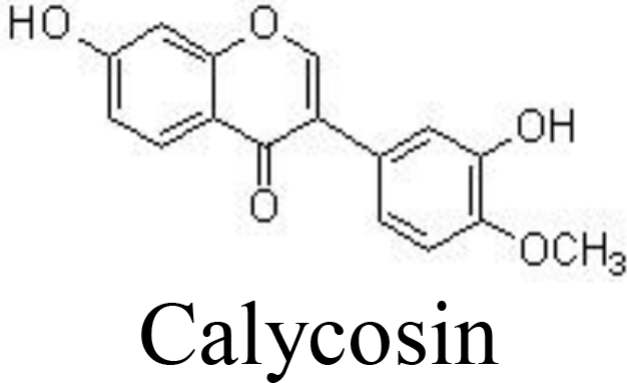 Calycosin	Primary mouse chondrocytes	100, 200, 400 μM	Inhibits IL-6, TNFα, iNOS, COX-2, MMP-3, and MMP-13; inhibits apoptosis; Inhibits NF-κB and PI3K/AKT pathways Increase collagen II and aggrecan	Shi et al. (95)
	DMM-induced mouse OA	40 mg/kg/day for 8 weeks; intraperitoneal injection		
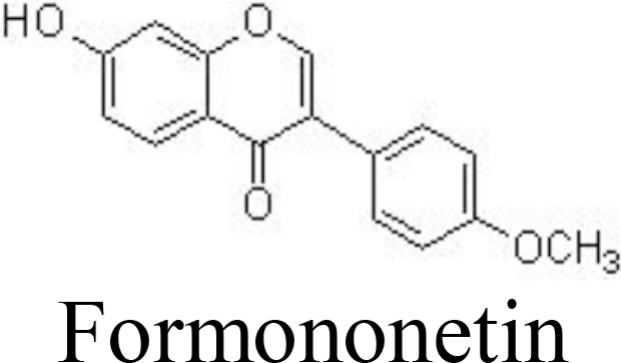 Formononetin	Primary rat chondrocytes	25, 50 μM	Decreases IL-1α, IL-1β, IL-6, and TNFα Inhibits MMP-1/-3/-13 Inhibits NF-κB and MAPK pathways	Cho et al. (96)

Casticin can be obtained from *Vitex trifolia* L. (Lamiaceae) and exhibits various biological effects, including anti-inflammatory. Casticin inhibits MMP-13 expression and reduces cartilage destruction in DMM-induced OA in mice. Casticin decreases pro-inflammatory cytokine production, suppresses oxidative stress, and inhibits the NF-κB pathway in IL-1β-treated ADTC5 cells (71) and in primary human OA chondrocytes (70). Consistently, fisetin and icariin are also reported to inhibit the phosphorylation of IKK and IκB, decrease the expression of HIF-2α, and ameliorate MMP- and ADAMTS-mediated ECM degradation (72, 97) (**Table 1**). Quercetin and its glucosides, including hyperoside (quercetin-3-O-galactoside) and rutin (quercetin-3-O-rutinoside) have been extensively reported for their health-benefiting effects on different diseases, such as OA. Specifically, quercetin and its glucosides may significantly ameliorate histopathological alterations, decrease the serum levels of IL-1β and TNFα, and suppress the expression of TLR4 and NF-κB (73, 98, 99) (**Table 1**) (**Figure 3**).

A combination of rutin with bromelain and trypsin in randomized controlled trials for treating patients with OA showed improvement in the Lequesne Algofunctional Index score and joint pain compared to patients receiving NSAIDs (100). A complex of glucosamine (1,200 mg/day), chondroitin (111 mg/day), and quercetin glucoside (45 mg/day) for 3 months has been reported to be effective in alleviating joint pain symptoms, increasing daily activity, and improving the properties of the synovial fluids in patients with OA. However, no beneficial effects are observed in patients with rheumatoid arthritis (101). Similarly, daily administration of complex tablets, including 45 mg of quercetin glycosides, 60 mg of chondroitin sulfate, and 1,200 mg of glucosamine hydrochloride, for 16 weeks may ameliorate aggregate scores and improve clinical symptoms in patients with OA, compared with those patients receiving dummy placebo tablets (102). In addition, isorhamnetin and morin also decrease ROS production, chondrocyte apoptosis, and the microenvironment in subchondral bone by inhibiting the NF-κB, MAPK, and AKT pathways (75, 76, 103) (**Table 1**) (**Figure 3**).

#### 3.1.2 Flavones

Acacetin (5,7-dihydroxy-4-methoxyflavone) and Linarin (Acacetin 7-O-rutinoside) may inhibit IL-1β-induced MMP-1, MMP-13, and MMP-13 expression by blocking NF-κB signaling in primary mouse chondrocytes and anterior cruciate ligament transection (ACLT)-induced OA in C57B/6 mice (77) and in human OA chondrocytes (104). Consistently, baicalin decreases the production of pro-inflammatory cytokines IL-6, IL-8, and TNFα, inactivates the NF-κB pathway, suppresses ECM degradation, and inhibits chondrocyte apoptosis (105, 106) (**Table 1**) (**Figure 3**). Flavocoxid, a medical food mainly containing two flavonoids, baicalin and catechins, exhibits protective effects by regulating the activity of arachidonic acid metabolism. It has been reported that flavocoxid (500 mg twice daily for 12 weeks) functions as effectively as naproxen (500 mg twice daily for 12 weeks) against patients’ knee OA, improving the clinical signs and symptoms (107, 108).

Similarly, luteolin and cymaroside (luteolin-7-O-glucoside) may decrease the levels of NO/iNOS, PGE_2_/COX-2, TNFα, MMP-1/-2/-3/-8/-9/-13, increase the production of collagen II, and inhibit the phosphorylation of p65 in IL-1β-treated rat chondrocytes (78, 109) (**Figure 3**). Morusin has also demonstrated anti-inflammatory activity against OA by inactivating the NF-κB pathway *in vivo* (40 mg/kg every 2 days by intragastric administration for 8 weeks) and *in vitro* (at the doses of 0.5, 1, and 2 μM) (110) (**Table 1**).

It has been reported that scutellarin, chrysin, and nobiletin may inhibit TNFα-induced inflammatory cytokines and ECM catabolic factors and enhance aggrecan and collagen II production by suppressing the NF-κB signaling pathway (79, 80, 111) (**Table 1**) (**Figure 3**). Similarly, jaceosidin and nepetin decrease the expression of MMP-3/-13 and ADAMTS-4/-5 in IL-1β-, IL-6-, or TNFα-treated mouse chondrocytes and DMM-induced mouse OA models by inhibiting NF-κB and MAPK pathways (81, 82) (**Table 1**). Endoplasmic reticulum (ER) stress has been associated with the activation of inflammation by activating the NF-κB pathway (112). Vitexin, an active compound from hawthorn leaf, has been demonstrated to inhibit ER stress, thereby inhibiting the NF-κB pathway and inflammatory responses (113). Wogonoside can ameliorate the histopathological changes and reduce the Mankins score in papain-induced rat OA models by inhibiting the NF-κB and ERK1/2 pathways (114).

#### 3.1.3 Flavanols

Epigallocatechin-3-gallate (EGCG), an active ingredient in green tea, has been linked to inflammation inhibition and cartilage degradation in OA. In IL-1β-treated human OA chondrocytes, EGCG targets to inhibit the levels of ENA-78, GM-CSF, GRO, GROα, IL-6/-8, MCP-1/-3, MIP-3α, MIP-1β, GCP2, IP-10, NAP-2, and LIF by inactivating NF-κB and JNK pathways in human OA chondrocytes (83), equine chondrocytes (115), and ATDC5 cells (116) (**Figure 3**). Silibinin is one of the main active compounds in the fruits and seeds of *Silybum marianum* L. (Asteraceae). Consistently, silibinin exhibits anti-inflammatory and bone-protective activity by downregulating the activity of PI3K/KAT and NF-κB pathways in human OA chondrocytes (84) (**Table 1**).

The maritime pine bark extract, Pycnogenol, has been standardized. Several clinical trials have been performed. In double-blind, randomized, placebo-controlled studies, Pycnogenol at concentrations of 100 mg/day and 150 mg/day for 3 months has been shown to ameliorate joint pain and stiffness and increase daily activity (117, 118). The clinical symptoms in the placebo group do not obviously change. In addition, Pycnogenol may decrease the dosage and frequency of NSAIDs or COX-2 inhibitors and reduce their adverse effects. The oral administration of Pycnogenol (100 mg twice daily for 3 weeks) has been reported to decrease the expression of MMP3, MMP-13, and ADAMTS-5 in patients’ serum (119).

#### 3.1.4 Favanones

Alpinetin, a flavonoid isolated from *Alpinia katsumadai* Hayata (Zingiberaceae), has shown various biological effects, including anti-inflammatory. It has been reported that alpinetin decreases the expression of MMP-13 and ADAMTS-5 and increases the expression of Col2a1, Bcl-2, and CKD1 by inhibiting NF-κB activation and stimulating ERK1/2 phosphorylation *in vivo* and *in vitro* (85). Eriodictyol is often found in citrus fruits and has reported broad bioactivities. Eriodictyol can decrease the levels of catabolic factors, such as NO/iNOS, PGE_2_/COX-2, and MMPs, by inactivating the NF-κB pathway and activating the NRF2/HO-1 pathway in IL-1β-treated human OA chondrocytes (86) (**Figure 3**). Similar results are also reached by naringenin, naringin, and pinocembrin (88, 120, 121) (**Table 1**).

Liquiritigenin is the main active compound from the rhizomes of *Glycyrrhiza uralensis* Fisch. (Leguminosae) and decreases IL-1β-induced expression of NO/iNOS, PGE_2_/COX-2, MMPs, and ADAMTSs in rat chondrocytes by inactivating NF-κB and MAPK pathways (87) (**Table 1**). Bavachin has been screened for interrupting DNA-binding activity, and bavachin (1, 2.5, 5, 10, and 20 μM) may decrease IL-1β-induced phosphorylation of IκBα and nuclear translocation of p65 and decrease the generation of chemokines in human chondrocytes and CHON-002 cells (122). Similarly, hesperetin inhibits IL-1β-induced inflammatory responses and ECM degradation by suppressing NF-κB and stimulating the NRF2 pathway in primary human chondrocytes and DMM-induced mouse OA models (89).

#### 3.1.5 Anthocyanins

The value of anthocyanins in protecting against the progression of OA and obesity has been comprehensively demonstrated (123). Cyanidin, one of the main anthocyanins, has been reported to have anti-inflammatory activity. In IL-1β-induced human OA chondrocytes, cyanidin and delphinidin suppress the production of NO/iNOS, PGE_2_/COX-2, IL-6, TNFα, MMP-13, and ADAMTS-5 and enhance the expression of aggrecan and collagen II by upregulating Sirt6 expression and downregulating the NF-κB pathway (90, 91) (**Table 1**). The methanolic purple corn extracts are rich in cyanidin-3-O-glucoside, pelargonidin-3-O-glucoside, and peonidin-3-O-glucoside. It has been demonstrated that purple corn extracts (6.25, 12.5, 25, and 50 μg/ml) exhibit anti-inflammatory activity against diabetes-mediated OA, as indicated by decreased AGE-induced release of glycosaminoglycan and expression of MMPs in human articular chondrocytes. The potential molecular mechanism might be associated with the inhibitory activity of anthocyanins in purple corn extracts against NF-κB and MAPK pathways (124, 125).

#### 3.1.6 Isoflavones

Genistein, a famous isoflavone in soybeans, has demonstrated anti-inflammatory and estrogen-like activities. In collagenase-induced rat temporomandibular joint OA (TMJOA) models, genistein can significantly improve the histopathological changes, reduce the levels of IL-1β and TNFα, and inhibit the expression of p65 (93) (**Table 1**). Biochanin A, isolated from *Trifolium pratense* L. (Leguminosae), has been shown to suppress IL-1β-induced inflammatory cytokines, such as NOS-2 and COX-2/PGE2, and MMP-1/-3/-13 and ADAMTS-5 expression by inhibiting the NF-κB signaling pathway in rat chondrocytes (94) and in rabbit chondrocytes and ACLT-induced rabbit OA models (126) (**Figure 3**). Similarly, calycosin, formononetin, and ononin (formononetin-7-O-glucoside) are reported to exhibit chondroprotective effects against inflammatory cytokines production, ECM degradation, and cell apoptosis by inhibiting the NF-κB signaling pathway *in vivo* and *in vitro* (95, 96, 127) (**Table 1**).

### 3.2 Flavonoids affects aging cells within OA by suppressing the NF-κB pathway

Aging, characterized by the accumulation of senescent cells and the resistance to apoptosis, is a risk factor for the development of various diseases and may increase the risk of hospitalization and death (128). Aging has become the primary risk factor for OA development. Chronic inflammation has been implicated in both OA development and the aging process. Potentially, targeting cellular aging has become a strategy to reverse the phenotype of OA chondrocytes (129). Chondrocyte senescence can be regulated by IL-1β. Silymarin has been shown to improve IL-1β-stimulated cell senescence, decrease catabolic gene expression, and restore chondrogenic phenotype factor expression (130). The senescence-associated secretory phenotype (SASP) is associated with the biological actions of senescent cells in producing inflammation-promoting factors. Procyanidin B2 (PCB2), comprised of two molecules of flavan-3-ol (−)-epicatechin, has been reported to ameliorate IL-1β-induced expression of SASP factors, inflammatory responses, and ECM degradation by mediating the NRF2 and NF-κB signaling pathways in rat chondrocytes (131). Similarly, Rhofolin exhibits significant effects against the expression of SASP factors and the phenotype of senescent cells by activating NRF2 signaling and suppressing the NF-κB pathway in IL-1β-treated chondrocytes (132). Malvidin has been shown to relieve joint pain, downregulate the expression of the apoptotic marker β-galactosidase, and decrease the production of IL-1β, IL-6, TNFα, and MMPs by inactivating the NF-κB pathway in MIA-induced rat OA models (92) (**Table 1**). Balcalein has been reported to ameliorate oxidative stress (133), which has been known to contribute to cell senescence and chondrocyte apoptosis. However, post-treatment of chondrocytes with baicalein does not improve the expression of SASP factors, although it may restore mitochondrial viability and suppress chondrocyte apoptosis by inhibiting the NF-κB pathway (134). Thus, the effects of natural flavonoids on OA chondrocyte senescence should be further elucidated.

### 3.3 The inhibitory effects of flavonoids against NF-κB signaling

Flavonoids have anti-inflammatory activity through several mechanisms, such as by interacting with related receptors and stimulating/inhibiting their activity, eliminating reactive oxygen/nitrogen species, suppressing the expression of inflammation-related factors, and inhibiting the secretion of cytokines. Some characteristics of flavonoid structures are critical for their anti-inflammatory effects: (1) a planar ring system; (2) the presence of C2=C3 or C4=O double bonds; (3) OH groups at C-5 and C-7 positions of the A ring; (4) OH groups at the B ring; (5) flavones and flavonols with an OH group at C-4’ of the B ring; (6) methoxy groups at C-3, C-5, or C-4’ positions; (7) flavones usually have a higher anti-inflammatory activity than the corresponding isoflavones, flavanols, and flavanones; (8) glycosides are often less active than their aglycones (135–137). The increased expression of COX2/PGE2, LOX, TXB2, and iNOS may be involved in the inflammatory responses. The suppressive activity of natural flavonoids with different structures, such as the different positions and numbers of the hydroxyl group, has been comprehensively discussed recently (137).

**Figure 4:**
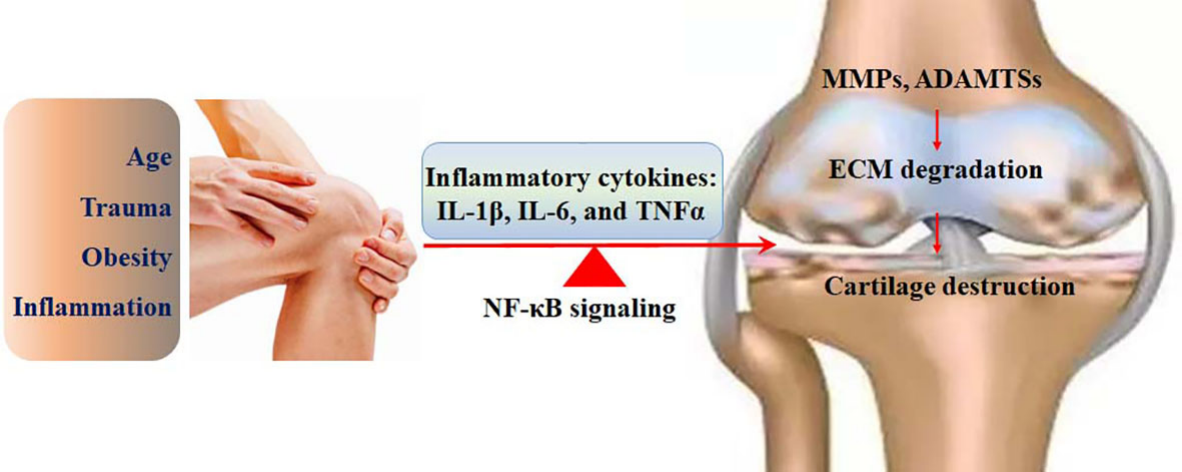
The involvement of the NF-κB signaling in the pathological development of OA. The risk factors, such as age, trauma, inflammation, and obesity, can activate the NF-κB signaling, which up regulates the expression of IL-1β, IL-6, and TNFα. The expression of catabolic enzymes, such as MMPs and ADAMTSs is enhanced by the NF-κB signaling, followed by ECM degradation and cartilage destruction.

Compared with diosmetin (flavone), hesperetin (flavanone) has a single C2=C3 bond without a C4=O double bond, and it has less activity against inflammation (138). Naringenin (flavanone) at the dose of 400 μM shows a similar efficacy against LPS-induced inflammation as apigenin (flavone) at the dose of 20 μM, suggesting an essential role for a C2=C3 double bond for anti-inflammatory activity (139). In LPS-stimulated IL-8 release, flavones, such as apigenin and luteolin, with a C2=C3 double bond in the C ring and an OH group at the C-5 and C-7 positions in the A ring, exhibit good anti-inflammatory activities. The deficiency of C2=C3 and/or C4=O double bonds in the C ring may lead to a reduction of anti-inflammatory activities (140) (**Figure 4**). However, these comparisons may be affected by different protocols and conditions.

The biological effects of flavonoids on inflammation might be affected by the number and position of substitutions. Hydroxyl groups in flavonoids may greatly contribute to their anti-inflammatory properties. It has been shown that C-6 and C-7 hydroxyl group substitutions in flavones may promote anti-inflammation, and the hydroxyl group at the C-8 position suppresses the activity of anti-inflammation (141). Quercetin (flavonol) has an OH group at the C-3 position, which is absent in luteolin (flavone). It has been reported that the IC_50_ values of quercetin on LPS-stimulated NO (62.4 μM) and COX-2 (72.3 μM) production are higher than those of luteolin on NO (14.26 μM) and COX-2 (59.9 μM) production (142). This indicates that the OH group at the C-3 position displays a negative effect on anti-inflammatory activity. Furthermore, genistein (an isoflavone) has a higher IC_50_ value (93.9 μM) on LPS-stimulated NO in RAW 264.7 macrophages compared to apigenin (14.24 μM) (142). Methoxylation of the OH group on a flavone often increases its anti-inflammatory activity. For example, quercetin has a 10-fold lower IC_50_ value of 2.4 μM than luteolin (143).

In TNFα-activated NF-κB signaling, 30 flavonoids were involved to explore the structure-activity relationship in suppressing NF-κB. A group with an electronegative property at C-5 of the A ring favors inactivating NF-κB through suppressing IKK activity. Similarly, a bulky or hydrophobic substituent at the meta position of the B ring also contributes to NF-κB inactivation. However, substitutions in C-8 of the A ring decrease its activity (144). Phosphorylation of IκBα contributes to the activation of NF-κB. One study demonstrated that the hydroxyl groups in C-5, C-6, and C-7 can effectively increase the anti-inflammatory activity of flavones by suppressing IκBα phosphorylation, while almost all the other groups are insensitive to the inhibition of IκBα phosphorylation (145). Flavonoids have been considered inhibitors of NF-κB signaling.

It has been reported that apigenin and genistein may interact with the IκBα/NF-κB complex with the binding energies of −34.0 and −31.7 kJ/mol, respectively, leading to decreased IκBα and p65 phosphorylation, attenuated NF-κB nuclear translocation, and inactivated NF-κB signaling (146). Similarly, quercetin, chrysin, pinocembrin, galangin, pinobanksin, and nobiletin can suppress NF-κB signaling by inhibiting IκBα and p65 phosphorylation and suppressing NF-κB nuclear translocation (147–149). Both cajanin (3’,5-dihydroxy-7-methoxy-isoflavone) and prunetin (5-hydroxy-7-methoxy-isoflavone) may inhibit IκBα and p65 phosphorylation. However, cajanin but not prunetin can suppress the nuclear translocation of NF-κB (150). Interestingly, apigenin, luteolin, and fisetin have been reported to inhibit the transcriptional activity of NF-κB but have not had any effects on IκBα degradation, p65 nuclear translocation, or p65-DNA binding (151). In addition, acetylation may promote the transcriptional activity of NF-κB, and Sirt1 can induce the acetylation of NF-κB (152). Fisetin has been reported to increase Sirt1 expression and decrease inflammatory responses in IL-1β-treated chondrocytes (72). Consistently, rutin protects articular chondrocytes against oxidative stress by activating Sirt1 expression and suppressing the NF-κB/MAPK signaling pathway in H_2_O_2_-treated chondrocytes (153).

## 4 Perspectives

Flavonoids are the most abundant polyphenols with health-beneficial activity in plants and foods. It is important for the food industry to supplement the aglycones, which have high absorption rates and plasma concentrations. Additionally, some therapeutic effects may be produced by the metabolites of these aglycones (154). Natural flavonoids have been explored as a therapeutic strategy to manage bone diseases such as OA. For example, Diosmetin exhibits protective activity against subchondral bone loss and cartilage degradation by decreasing the MAPK signaling pathway in RANKL-treated bone marrow-derived monocytes and DMM-induced mouse OA models (155). Baicalein has been shown to protect against OA development by enhancing the expression of the AMPK/NRF2/HO-1 signaling pathway and reducing chondrocyte ferroptosis (156). In addition, intra-articular injection of galangin exhibits chondroprotective effects against oxidative stress and ECM degradation by activating proline/arginine-rich and leucine repeat protein (PRELP) expression in human OA chondrocytes (157). Similarly, the overproduction of inflammatory cytokines and ECM-catabolic factors can be ameliorated by formononetin *via* mediating PTEN/AKT/NF-κB signaling in IL-1β-treated human chondrocytes (158).

The effectiveness of the flavonoids discussed above has been demonstrated. However, the therapeutic efficacy in managing complex and chronic diseases, such as OA, by employing an individual candidate may be limited. Probably, a combination with other drugs may provide an effective approach. Disappointingly, information about this strategy is rather limited. Although there are multiple beneficial pharmacological effects of flavonoids, studies on the therapeutic efficacy of flavonoids obtained from various resources in human beings are still needed. It is crucial to note that flavonoids should be supplemented with caution, particularly those that may produce food–drug interactions and untoward reactions. In addition, useful strategies should be developed for increasing the efficiency of tissue-target delivery, enhancing bioavailability, and improving the therapeutic effects, although structural modifications of flavonoids have already been highlighted (159). Recently, gut microbiota-regulated metabolism has been implicated in various fields. Whether it poses an effect on the pharmacology of flavonoids still needs for further investigation.

Great progress has been made in studying the pharmacological roles of natural flavonoids and their significance in the therapeutic management of OA. However, more exploration of the microbial metabolism of flavonoids is still needed due to their limited absorption characteristics and gut microbiome-regulated degradation in the colon. Potentially, the microbial metabolites of flavonoids may be the effective compounds responsible for the pharmacological actions of the parent flavonoids. The interaction between the gut microbiome and natural flavonoids should be included in the evaluation when exploring flavonoids to therapeutically manage OA. Thus, future investigations of OA in the exploration of new potential drugs may act on more than one target, which would exhibit a positive/negative effect on OA treatment. The underlying mechanisms of OA development are rather complicated, and they are the rational basis for new drug development. Most clinical pharmacotherapies available for OA treatment are symptomatic. For instance, the role of IL-1β in the pathological development of OA has been demonstrated to be a target. An animal investigation using an IL-1 receptor antagonist has reported promising results. However, its biological effects on OA patients still need further investigation. More efficient inflammatory biomarkers for predicting OA progression and treatment are needed to be further explored, and more potential drug targets are also needed to be discovered.

## 5 Conclusion

**Figure 5:**
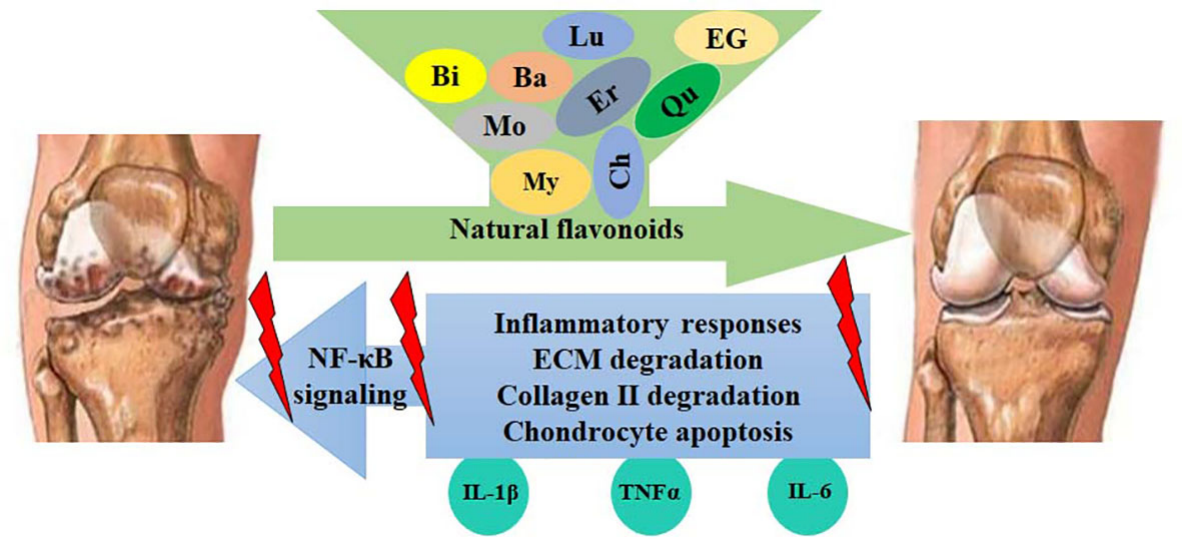
Flavonoids protect against OA development by inhibiting the NF-κB-mediated inflammatory responses. Activated NF-κB signaling increases the expression of IL-1β, TNFα, COX-2, PGE2, iNOS, NO, MMPs, and ADAMTSs, leading to the enhancement of ECM degradation, collagen II degradation, and chondrocyte apoptosis. These catabolic responses can be blocked by flavonoids, such as myricetin (My), quercetin (Qu), morin (Mo), baicalin (Ba), luteolin (Lu), chrysin (Ch), EGCG (EG), eriodictyol (Er), and biochanin A (Bi).

OA is characterized by low-grade chronic inflammation, and the inflammatory responses greatly promote the pathological changes and progression of OA. Anti-inflammatory therapy has become an effective strategy for the therapeutic management of OA. The NF-κB signaling pathway plays a crucial role in inflammatory actions, which contribute to chondrocyte injury and ECM degradation. Many inflammatory cytokines, such as IL-6 and TNFα, and ECM-degrading enzymes, such as MMPs and ADAMTSs, are transcriptional targets of the NF-κB pathway. Increased NF-κB pathway activity is associated with the pathological changes of OA, and targeting the NF-κB pathway has become an effective therapeutic strategy. Flavonoids, the most abundant natural polyphenols, have been reported to have multiple pharmacological effects, particularly anti-inflammatory activity. A large body of research indicates that natural flavonoids protect against OA development by inactivating the NF-κB pathway, reducing the levels of inflammatory cytokines, and inhibiting the degradation of ECM (**Figure 5**). However, most studies focus on individual flavonoid compounds in protection against OA, which may have limited therapeutic efficacy. Additionally, clinical trials of natural flavonoids for humans are still rather rare. More efforts are still needed.

## Author contributions

JZ: Conceptualization and methodology. JZ and YY: Data curation, writing-original draft preparation, data curation, validation, and writing-reviewing and editing. All authors contributed to the article and approved the submitted version.
